# The Comparative Effect of Immediate and Delayed Feedback on EFL Learners' Engagement and Willingness to Collaborate

**DOI:** 10.1002/pchj.70046

**Published:** 2025-08-23

**Authors:** Hongyu Mai

**Affiliations:** ^1^ School of Foreign Languages Guangxi University Nanning China; ^2^ Asia‐Pacific (Southeast Asia) Institute for Translation and Intercultural Studies Nanning China

**Keywords:** delayed feedback, EFL learners, engagement, immediate feedback, willingness to practice collaborative learning

## Abstract

This study examines the comparative effects of immediate and delayed feedback on the engagement and willingness to participate in collaborative learning of English as a Foreign Language (EFL) learners. Ninety EFL students at Guangxi University participated in a quasi‐experimental study across three conditions: no feedback, immediate feedback, and delayed feedback. Using one‐way ANOVA, the results revealed that both feedback types significantly influenced learners' engagement across affective, cognitive, and behavioral dimensions, with immediate feedback yielding significant effects in the affective and cognitive domains. Delayed feedback, however, was more effective in fostering willingness to collaborate, likely due to the reflective space it provided. These findings suggest that the timing of feedback plays a crucial role in shaping learner outcomes and should be strategically aligned with instructional goals. The study highlights the importance of context‐sensitive feedback practices, particularly in digital learning environments where timing constraints and student autonomy vary.

## 1 Introduction

Within the cognitive‐integrationist research paradigm, scholars in second language acquisition (SLA) broadly agree that corrective feedback (CF) plays a crucial role in facilitating the acquisition of a second language (Canals et al. 2020). This consensus is supported by various meta‐analyses, including those conducted by Goo and Mackey (2013) and a comprehensive review by Nassaji (2009). These analyses consistently demonstrate that CF has a medium to large effect size on second language (L2) learning. The theoretical underpinning for employing CF is derived from the Interaction Hypothesis, which asserts that conversational interaction enhances L2 learning by rendering linguistic input more comprehensible. Furthermore, CF during conversational interaction is posited to contribute to L2 learning by drawing learners' attention to formal language aspects and triggering cognitive processes such as noticing.

Corrective feedback (CF) constitutes a pivotal aspect of English as a foreign language (EFL) and English as a second language (ESL) classrooms. Numerous studies have explored the categorization of CF strategies and their effects on L2 development, grounded in various theoretical perspectives including cognitive theory, skill‐learning theory, and sociocultural theory (Fu and Li 2022). The substantial interest in CF reflects its dual importance: its role in advancing SLA theory and its pedagogical relevance to classroom instruction (Hiver et al. 2020).

It is imperative to recognize, however, that CF alone does not result in language acquisition. Feedback functions as a dynamic, dialogic process requiring active learner engagement. Its effectiveness is contingent upon how learners interpret and respond to it during instruction (Hanley 2023). Understanding learners' responsibility for, and receptiveness to, feedback is therefore essential for ensuring its positive impact (Zhang and Hyland 2018; Zhang 2021).

Wiliam's (2011) assertion that “feedback should be more work for the recipient than the donor” underscores the centrality of learner engagement. This notion underscores the importance of examining how cognitive, behavioral, and affective engagement impact learners' perceptions of and responses to feedback. Price et al. (2011) highlight the influence of engagement on feedback efficacy, while (Shen and Chong 2023) advocate for personalized approaches that consider learners' unique motivations and reactions to feedback.

Despite growing recognition of the role of engagement, empirical studies explicitly linking different types of classroom feedback (CF) to measurable outcomes in EFL/ESL learners' engagement remain limited. To our knowledge, few studies have systematically examined how the timing of CF—immediate versus delayed—affects learners' multidimensional engagement in classroom settings. Furthermore, although prior research has explored CF's effects on individual language skills and willingness to communicate, its specific influence on learners' willingness to engage in collaborative learning remains underexplored.

In light of these gaps, the present quasi‐experimental study aims to investigate the effects of feedback timing on learner engagement and collaborative behavior in EFL contexts. Specifically, it explores the interrelationship between the timing of CF and learners' affective, cognitive, and behavioral engagement, as well as their willingness to participate in collaborative learning activities.

Theoretical assumptions support the role of CF in facilitating L2 acquisition, particularly in drawing learners' attention to linguistic form and meaning. Broadly defined, CF includes “comments or other information that learners receive concerning their success on learning tasks or tests, either from the teacher or other persons” (Lyster and Ranta 2013). In their framework, oral feedback is categorized into input‐providing and output‐prompting types and further distinguished by implicitness and explicitness. Specific strategies include explicit correction, recast, repetition, elicitation, metalinguistic clues, and clarification requests. For written CF, feedback is classified into two types: direct and indirect, as well as metalinguistic and non‐metalinguistic. This comprehensive categorization underlines CF's potential in bridging the gap between learner output and target norms (Han 2019).

While the effectiveness of CF across formats has been widely studied, *the role of timing in optimizing learner engagement and collaborative orientation has received insufficient empirical attention*. This study addresses that need through the following research questions:

1. Does the timing of feedback (immediate or delayed) significantly impact EFL learners' engagement in the classroom?
2. Does the timing of feedback (immediate or delayed) significantly impact EFL learners' willingness to practice collaborative learning?

## 2 Literature Review

### 2.1 Theoretical Framework

This study adopts an integrated framework combining the Interaction Hypothesis, Skill Acquisition Theory (DeKeyser et al. 2017), and the Engagement Framework (Hiver et al. 2020) to explain the role of CF in shaping L2 learner engagement. These theories were selected for their complementary insights into how learners notice, internalize, and respond to feedback. The Interaction Hypothesis highlights the role of CF in promoting linguistic noticing during communication. Skill Acquisition Theory accounts for the gradual proceduralization of accurate language forms through feedback and repeated practice. The Engagement Framework emphasizes the emotional, cognitive, and behavioral dimensions of learner involvement in feedback processes. Together, these perspectives provide a coherent framework for analyzing how feedback timing affects various aspects of learner engagement in classroom settings.

### 2.2 Learner Engagement and Corrective Feedback

Learner engagement is widely regarded as a multidimensional construct encompassing affective, cognitive, and behavioral components (Guilloteaux 2016; Han and Hyland 2015). Cognitive engagement encompasses learners' metacognitive operations in processing feedback, including noticing, understanding, and applying corrections. Affective engagement refers to learners' emotional responses, including interest, frustration, or appreciation toward feedback. Behavioral engagement encompasses observable actions, such as task completion and revision efforts in response to feedback on changes (Han 2017; Moser 2020).

Several researchers have underscored the mediating role of engagement in the effectiveness of CF. According to Moser (2020), learner engagement with CF is influenced by both internal and external mediators, including attitudes, emotions, and social context. This is consistent with Han's (2019) ecological perspective, which frames feedback as part of a complex interaction between learners and their environments.

Studies show that learners' engagement with CF is shaped by individual factors, such as self‐efficacy and language proficiency, as well as by the quality of feedback provided (Shen and Chong 2023; Vattøy and Smith 2019). Zheng and Yu (2018), for example, reported that lower‐proficiency learners demonstrated reduced cognitive and behavioral engagement with written CF. Similarly, found that engagement levels were moderated by learners' beliefs, expectations, and the perceived clarity of the feedback.

Emotional factors also play a critical role in mediating feedback effects. Manhood (2017) and Enel and Yao (2021) observed that learners' affective reactions to feedback can either support or hinder engagement, depending on whether the feedback is perceived as supportive or evaluative. Moser (2020) further emphasized that the presence of clear guidance and a supportive classroom climate enhances affective engagement, especially when learners are encouraged to view feedback as a resource rather than a judgment.

Taken together, the literature suggests that learner engagement with CF is not uniform but varies across cognitive, affective, and behavioral domains, and is influenced by a constellation of personal and contextual variables (Shen and Chong 2023).

### 2.3 Corrective Feedback and Collaborative Learning

Corrective feedback has also been linked to learners' willingness to engage in collaborative learning, a key competency in 21st‐century education (OECD 2017; Pellegrino and Hilton 2012). Collaborative learning environments foster mutual support, peer interaction, and the co‐construction of knowledge, which are considered essential for both academic and social development (Gillies 2008; Shonfeld et al. 2017).

The effectiveness of CF in collaborative contexts depends on its ability to foster autonomy, reflection, and shared responsibility. Zhang (2021) reported that well‐designed feedback processes can enhance learners' readiness to engage with peers, particularly when feedback promotes dialog and goal alignment. Weinberger and Shonfeld (2020) also found that delayed feedback allowed students to reflect more deeply, increasing their motivation to contribute to group tasks.

However, challenges remain in implementing collaborative learning in higher education. Despite the documented benefits of cooperative approaches (Cockerill et al. 2018; Henderson et al. 2018; Pellegrino and Hilton 2012), traditional instructional models continue to dominate, often emphasizing individual performance and limiting opportunities for interdependent learning. Research suggests that when collaboration is implemented only at basic levels, such as peer review or surface discussion, it may not fully leverage the potential benefits of group interaction (Johnson et al. 2014).

Teachers play a pivotal role in facilitating effective collaboration by providing clear roles, encouraging active participation, and integrating feedback as a formative component of group learning (Harasim 2012).

While existing studies highlight the importance of learner engagement and its relationship with corrective feedback, limited attention has been given to how the timing of feedback—immediate versus delayed—affects engagement across its three dimensions and learners' willingness to collaborate. This study aims to fill that gap by examining how feedback timing influences both individual engagement and collaborative dispositions in an EFL context, drawing on a unified theoretical framework to interpret the results.

## 3 Methodology

### 3.1 Study Design and Participants

In a quasi‐experimental study conducted at the School of Foreign Languages, Guangxi University, three intact classes comprising 90 learners of English for academic purposes were selected through convenience sampling. The participants, aged between 21 and 27, were exclusively native speakers of the Chinese language, with 40% being male and 60% female. All participants were undergraduate students specializing in various engineering fields. Although convenience sampling was used, random assignment of the intact classes to experimental conditions was implemented to support internal validity. To ensure group equivalence, participants' pre‐test scores on both engagement and willingness to collaborate were compared using ANOVA, which confirmed that there were no significant baseline differences across the groups (*p* > 0.05). Each intact group was strategically assigned to one of three feedback conditions: no feedback, immediate feedback, and delayed feedback. Employing a repeated measures research design, the study aimed to investigate the impact of different feedback conditions on language learning outcomes. The adapted questionnaires were administered to all participants at the onset and conclusion of the treatment, providing a comprehensive understanding of the potential effects of feedback timing on language acquisition within the unique context of English for academic purposes instruction at Guangxi University.

### 3.2 Instrumentation

The research employed two distinct instruments to collect data at the commencement and culmination of the treatment. Firstly, the willingness to practice collaborative learning was assessed using an instrument that was developed by Weinberger and Shonfeld (2020). Comprising seven items, this instrument assessed participants' receptiveness and inclination toward collaborative learning practices. Willingness was operationally defined as learners' reported likelihood of participating in group‐based language learning activities, rated on a five‐point Likert scale. Secondly, Instrument B, the Student Engagement Scale, served as a self‐report measure to evaluate engagement in classroom activities. This scale, encompassing three dimensions—Affective Enjoyment, Cognitive Engagement, and Behavioral Engagement—yielded a total score ranging from 12 to 60, with higher scores indicative of heightened engagement. Engagement was operationalized as the composite of affective (e.g., interest/enjoyment), cognitive (e.g., mental effort), and behavioral (e.g., participation) responses to instructional activities.

The data collection process was further enriched by semi‐structured interviews conducted with participants in the experimental group, guided by an interview checklist (Cohen et al. 2002). The checklist featured open‐ended questions and prompts crafted to solicit participants' perceptions and experiences of the intervention. Uniquely designed for this study, the interview checklist underwent a rigorous validation process involving expert review and confirmation by three colleagues well‐versed in qualitative research methods. Thematic analysis was conducted using open and axial coding, with NVivo software employed to organize, categorize, Furthermore, the emerging patterns related to learners' perceptions of feedback were analyzed. Cronbach's alpha demonstrated the reliability of the estimates from both instruments: affective engagement α = 0.84, cognitive engagement α = 0.86, behavioral engagement α = 0.85, and willingness α = 0.83, confirming the robustness and reliability of the data collection tools used in this study.

### 3.3 Feedback Treatment

This study involved three groups who performed summary retelling tasks on the Sky room app following the steps previously described. However, the three groups differed in terms of when they received CF. Participants in the immediate CF group received CF while performing the task (online CF). Learners in the short‐term CF group received it after completing the task, and the third group, the delayed CF group, received it a few days later in the following online extended session (offline CF). The type of feedback used in summary retelling tasks was a combination of repeating the learner's error and providing an explicit correction. A repetition of an error was chosen to precede the explicit correction to make the CF move more salient; that is, an erroneous production was repeated with the error highlighted via a prosodic emphasis to encourage self‐correction and is followed by an explicit correction. This hybrid CF, as Henderson et al. (2018) argues, must be effective and salient to L2 learners and is frequently employed by L2 teachers. Li (2018) assumes that this type of CF move can facilitate L2 development in different ways because reformulation is more effective for learning new linguistic targets while prompts are better at consolidating previously learned items.

The CF was implemented as follows: For the immediate CF group, the teacher repeated the erroneous sentence with prosodic emphasis and paused for approximately 5 s to allow the learner to self‐correct. If the participant could self‐correct, the instructor would approve and point to him to continue. However, if the participant still made the same error or there was no response, the teacher repeated the entire erroneous utterance and then explicitly corrected it, highlighting the correct form for the learner. For instance, if a learner says, “The cake is eaten yesterday,” the teacher replies, “The cake was eaten yesterday?” He paused for about 5 s for self‐correction. If the learner continued committing the same error, the teacher would then explicitly correct the sentence as “The cake was eaten yesterday.” It is worth mentioning that correcting the erroneous vocabulary or morpho‐syntax was done slowly and emphatically. The same error correction procedure was followed for other study groups, except that the participants of the second and third groups received CF after finishing the target tasks in the classroom and a few days later (in the following online session), respectively.

### 3.4 Procedure

This research study followed a systematic procedure that unfolded in multiple steps. Initially, the researcher obtained consent from the relevant authorities to conduct the study at the specified language institute. Subsequently, each intact class was assigned to one of three treatment conditions: the immediate corrective feedback (CF) group (Group 1), the no feedback group (Group 2), and the delayed CF group (Group 3). To enhance internal validity despite using intact classes, random assignment was applied at the class level and supported by pretest equivalence checks. The instructional sessions were conducted face‐to‐face and took place approximately ninety minutes a day, twice a week. The three intact classes received the adapted instruments at the onset of the study. The subsequent phase involved three treatments implemented for all groups during instructional sessions.

Although the three groups received the same materials and instructions, the timing of CF varied. Group 1 received immediate CF during each summary retelling task, Group 2 received no feedback during the session, and Group 3 was reminded of their errors in the next session before receiving CF. This procedure was consistently applied throughout the treatment. Upon completion of the treatment, an immediate posttest was administered to all groups 2 days after the treatment concluded. Additionally, immediate and delayed posttest scores were compared to assess whether the different feedback timings had equivalent effects on their engagement and willingness. All steps were meticulously recorded through audio and video means, transcribed, and strictly coded for subsequent in‐depth analysis, with careful attention to ensuring equal performance times for the three groups during the treatment.

### 3.5 Data Analysis

To assess the impact of feedback timing on engagement and willingness to participate in collaborative learning, a one‐way analysis of variance (ANOVA) was conducted. The independent variable was the type of feedback, categorized into three levels: no feedback, delayed feedback, and immediate feedback. The dependent variables were participants' reported engagement and willingness to participate. Before conducting the ANOVA, assumptions were checked, including normality and homogeneity of variances. No violations were observed, justifying the use of a one‐way ANOVA for comparing means across the three groups.

Post hoc tests using the Bonferroni correction were conducted to further explore these differences. The Bonferroni method was selected over other post hoc tests (e.g., Tukey HSD) due to its conservative nature, which provides stricter control of the familywise error rate—especially appropriate when making multiple pairwise comparisons with unequal sample variances or when minimizing Type I errors is prioritized. Post hoc comparisons for engagement and willingness to participate are presented below.

### 3.6 Ethical Considerations

Ethical approval for the research procedures was obtained from the Ethics Committee of Guangxi University.

Prior to data collection, all participants were informed about the study's objectives, their voluntary participation, the confidentiality of their responses, and their right to withdraw at any time without penalty. Written informed consent was obtained from all participants (or from legal guardians in the case of minors). The study adhered to the principles outlined in the Declaration of Helsinki.

## 4 Results

### 4.1 Research Question One

This study aimed to investigate the effect of feedback timing on the engagement of EFL learners, with a specific focus on the distinct types of engagement—Affective, Cognitive, and Behavioral. Table 1 presents the results of a one‐way ANOVA conducted to assess the homogeneity of engagement across the three feedback timing conditions (no feedback, immediate feedback, and delayed feedback) for each type of engagement: Affective, Cognitive, and Behavioral.

**TABLE 1:** One‐way ANOVA for the groups' engagement before the test.

Engagement type	Group	*N*	Mean	SD	*F*	*p*	*η* ^2^
Affective	No feedback	30	3.01	0.36	0.87	> 0.05	0.01
	Immediate feedback	30	2.90	0.39			
	Delayed feedback	30	2.93	0.39			
Cognitive	No feedback	30	3.01	0.36	0.91	> 0.05	0.08
	Immediate feedback	30	2.90	0.39			
	Delayed feedback	30	2.97	0.19			
Behavioral	No feedback	30	3.01	0.36	1.00	> 0.05	0.09
	Immediate feedback	30	2.90	0.39			
	Delayed feedback	30	3.02	0.32			
Engagement	No feedback	30	3.01	0.36	0.96	> 0.05	0.11
	Immediate feedback	30	2.90	0.39			
	Delayed feedback	30	2.99	0.27			

As shown in Table 1, the one‐way ANOVA yielded an *F*‐value of 0.87 (*p* > 0.05) for Affective Engagement, indicating no statistically significant differences among the three groups. Mean scores were comparable across the no‐feedback group (*M* = 3.01, SD = 0.36), immediate‐feedback group (*M* = 2.90, SD = 0.39), and delayed‐feedback group (*M* = 2.93, SD = 0.39). Similarly, for Cognitive Engagement, the ANOVA produced an *F*‐value of 0.91 (*p* > 0.05), with mean scores remaining consistent across the no‐feedback (*M* = 3.01, SD = 0.36), immediate‐feedback (*M* = 2.90, SD = 0.39), and delayed‐feedback (*M* = 2.97, SD = 0.19) groups.

For Behavioral Engagement, the ANOVA resulted in an *F*‐value of 1.00 (*p* > 0.05), again revealing no significant differences, as mean scores were similar across the no‐feedback (*M* = 3.01, SD = 0.36), immediate‐feedback (*M* = 2.90, SD = 0.39), and delayed‐feedback (*M* = 3.02, SD = 0.32) conditions. The analysis of Overall Engagement, combining all three components, also showed no significant effect of feedback timing, with an *F*‐value of 0.96 (*p* > 0.05). Mean scores for overall engagement were nearly identical across the no‐feedback (*M* = 3.01, SD = 0.36), immediate‐feedback (*M* = 2.90, SD = 0.39), and delayed‐feedback (*M* = 2.99, SD = 0.27) groups.

The consistent lack of significant *F*‐values across all engagement domains suggests that, at this preliminary stage, feedback timing did not differentially affect the engagement levels of EFL learners. This uniformity points to a degree of baseline homogeneity among the groups in their affective, cognitive, and behavioral responses. These findings provide a foundation for subsequent analyses that aim to explore more nuanced or delayed effects of feedback timing on learner engagement. Further investigation, including post hoc comparisons, will help clarify whether specific engagement domains are more sensitive to immediate or delayed feedback conditions. Results are detailed in Table 2.

**TABLE 2:** ANOVA tests for comparing the groups' engagement after the treatment.

Engagement type	Group	*N*	Mean	SD	*F*	*p*	*η* ^2^
Affective	No feedback	30	3.08	0.29	84	0.000	0.73
	Immediate feedback	30	4.16	0.30			
	Delayed feedback	30	3.73	0.36			
Cognitive	No feedback	30	3.10	0.264	91.26	0.000	0.74
	Immediate feedback	30	4.18	0.306			
	Delayed feedback	30	3.73	0.34			
Behavioral	No feedback	30	3.17	0.22	91.36	0.000	0.75
	Immediate feedback	30	3.73	0.30			
	Delayed feedback	30	3.90	0.36			
Engagement	No feedback	30	3.10	0.22	96.23	0.000	0.70
	Immediate feedback	30	4.12	0.30			
	Delayed feedback	30	3.65	0.35			

As illustrated in Table 2, the analysis of variance (ANOVA) for Affective Engagement revealed a statistically significant effect among the groups, *F*(2, 114) = 84.00, *p* < 0.001, with a large effect size (*η* ^2^ = 0.73), indicating strong practical significance. Mean scores varied across conditions: no‐feedback (*M* = 3.08, SD = 0.29), delayed‐feedback (*M* = 3.73, SD = 0.30), and immediate‐feedback (*M* = 4.16, SD = 0.36). These differences suggest that feedback timing significantly influenced affective engagement, with immediate feedback yielding the highest mean.

Similarly, for Cognitive Engagement, the one‐way ANOVA indicated a significant effect among the groups, *F*(2, 114) = 91.26, *p* < 0.001, with a large effect size (*η* ^2^ = 0.74). Mean scores were no‐feedback (*M* = 3.10, SD = 0.26), delayed‐feedback (*M* = 3.73, SD = 0.31), and immediate‐feedback (*M* = 4.18, SD = 0.34), suggesting that feedback type significantly shaped cognitive engagement. Post hoc analyses are warranted to identify specific differences between groups.

For Behavioral Engagement, the ANOVA results also showed a significant group effect, *F*(2, 114) = 91.36, *p* < 0.001, supported by a substantial effect size (*η* ^2^ = 0.75). Mean scores were no‐feedback (*M* = 3.17, SD = 0.22), immediate‐feedback (*M* = 3.73, SD = 0.30), and delayed‐feedback (*M* = 4.16, SD = 0.36), indicating the type of feedback significantly impacted behavioral engagement, with delayed feedback yielding the highest score. Again, post hoc tests are necessary for clarification.

Lastly, the analysis of Overall Engagement yielded a significant group effect, *F*(2, 114) = 96.23, *p* = 0.000, with a large effect size (*η* ^2^ = 0.74). Group means were: no‐feedback (*M* = 3.10, SD = 0.22), delayed‐feedback (*M* = 3.73, SD = 0.30), and immediate‐feedback (*M* = 4.17, SD = 0.35). These findings reinforce the role of feedback timing in shaping learners' holistic engagement levels. Results of the Bonferroni post hoc comparisons are provided in Table 3.

**TABLE 3:** Bonferroni tests for locating the sources of differences among the groups.

Dependent variable	(I) groups	(J) groups	Mean difference (I‐J)	*p*
Affective	No feedback	Immediate feedback	−0.64	0.000
		Delayed feedback	−0.433	0.000
	Immediate	Delayed feedback	0.642	0.000
Cognitive	No feedback	Immediate feedback	−0.62	0.000
		Delayed feedback	−1.07	0.000
	Immediate	Delayed feedback	−0.450	0.000
Behavioral	No feedback	Immediate feedback	−0.613	0.000
		Delayed feedback	−1.05	0.000
	Immediate	Delayed feedback	−0.436	0.000
Engagement	No feedback	Immediate feedback	−0.625	0.000
		Delayed feedback	−1.065	0.000
	Immediate	Delayed feedback	−0.440	0.000

### 4.2 Research Question 2

The second research question investigated the impact of feedback timing on the willingness of EFL learners to participate in collaborative learning. The results of the ANOVA tests comparing the scores of the three groups on willingness to participate in collaboration at the onset and end of the semester are presented in Table 4.

**TABLE 4:** ANOVA tests for groups' scores on willingness to participate in collaborative learning before and after the treatment.

		*N*	Mean	SD	*F*	*P*	*η* ^2^
Before	No feedback	30	3.2	0.37	0.87	> 0.05	…
	Immediate feedback	30	3.1	0.41			
	Delayed feedback	30	3.23	0.38	.		
After	No feedback	30	3.33	0.39	87	< 0.05	0.86
	Immediate feedback	30	3.87	0.42			
	Delayed feedback	30	4.25	0.20			

As shown in Table 4: ANOVA tests for groups' scores on willingness to participate in collaborative learning before and after the treatment, the pre‐treatment analysis revealed no statistically significant differences in willingness to participate (WTP) among the no‐feedback (*M* = 3.20, SD = 0.37), immediate‐feedback (*M* = 3.10, SD = 0.41), and delayed‐feedback (*M* = 3.23, SD = 0.38) groups, *F*(2, 87) = 0.87, *p* > 0.05, indicating that the groups were comparable before the intervention. In contrast, post‐treatment results revealed a statistically significant difference among the three groups, *F*(2, 87) = 87.00, *p* < 0.05, with a large effect size (*η* ^2^ = 0.86). Mean scores increased in all groups, with the no‐feedback group at *M* = 3.33 (SD = 0.39), the immediate‐feedback group at *M* = 3.87 (SD = 0.42), and the delayed‐feedback group showing the highest mean at *M* = 4.25 (SD = 0.20), suggesting that feedback—particularly delayed feedback—enhanced learners' willingness to engage in collaborative learning.

Bonferroni tests (Table 5) for locating the sources of differences among the groups' WTP for collaborative learning confirm that all pairwise comparisons were statistically significant. Participants in the immediate‐feedback group reported significantly higher WTP scores than those in the no‐feedback group (mean difference = −0.54, *p* = 0.000), while those in the delayed‐feedback group scored significantly higher than both the no‐feedback (mean difference = −0.94, *p* = 0.000) and immediate‐feedback groups (mean difference = 0.50, *p* = 0.000). These findings underscore that both feedback timing conditions had a positive impact, but delayed feedback was particularly effective in enhancing learners' willingness to collaborate.

**TABLE 5:** Bonferroni tests for locating the sources of differences among the groups' WTP for collaborative learning.

Dependent variable	(I) groups	(J) groups	Mean difference (I‐J)	*p*
WTP CL	No feedback	Immediate feedback	−0.54	0.000
		Delayed feedback	−0.94	0.000
	Immediate	Delayed feedback	0.50	0.000

## 5 Discussion

The primary aim of this study was to investigate how the timing of feedback—immediate versus delayed—affects EFL learners' engagement and their willingness to participate in collaborative learning. The results revealed significant effects of feedback timing on all engagement dimensions (affective, cognitive, and behavioral) as well as overall engagement, with large effect sizes, indicating that these differences were not only statistically significant but also practically meaningful.

Immediate feedback was found to be particularly effective in enhancing affective and cognitive engagement. This aligns with prior research, which shows that timely, focused feedback supports motivation, attention, and conceptual reinforcement in second language acquisition (Fu and Li 2022; Han 2019). In contrast, delayed feedback, while still beneficial, was relatively less impactful in these areas. This temporal sensitivity underscores the importance of aligning feedback delivery with instructional moments to support learner processing and affective response.

Notably, the present study found that even behavioral engagement—often considered more task‐driven and resistant to instructional manipulation—was significantly influenced by the timing of feedback. This finding stands in contrast to previous work, such as Sato and McDonough (2019), which reported limited effects of feedback on behavior during interaction‐focused speaking tasks. A possible explanation for this divergence is the present study's emphasis on structured, scaffolded tasks following feedback, which may have amplified its influence on learners' observable behaviors. Such structured follow‐up activities, as noted by Gillies (2016), are essential for transferring engagement from internal reflection to overt classroom actions.

From a theoretical perspective, Skill Acquisition Theory (DeKeyser et al. 2017) helps explain the superior outcomes associated with immediate feedback. Prompt correction is likely to prevent the proceduralization of errors and support the automation of accurate forms through repeated practice. However, the effectiveness of delayed feedback—especially in enhancing collaborative learning—suggests additional dynamics at play. As seen in Canals et al. (2020), delayed feedback can promote self‐evaluation and autonomy by giving learners time to reflect, reprocess, and monitor their performance. In this study, delayed feedback may have functioned as a metacognitive trigger, encouraging deeper processing and self‐regulation.

This is particularly evident in the results on learners' willingness to participate in collaborative learning, where delayed feedback outperformed immediate feedback. This finding is consistent with studies such as Zhang (2021) and Weinberger and Shonfeld (2020), which emphasize that learner autonomy, reflection time, and reduced performance anxiety can enhance collaborative engagement. Delayed feedback might have lowered the pressure associated with immediate evaluation, allowing students to critically revisit their output and draw connections with peer input, principles supported by Social Interdependence Theory (Cockerill et al. 2018; Johnson et al. 2014).

Best et al. (2015) also noted that ESL learners often express a preference for feedback that allows time for consideration and revision, aligning with the affective benefits observed in this study. Moreover, this supports the constructivist perspective (Burr 2015), which argues that learners co‐construct knowledge through engagement, reflection, and dialogue over time, rather than through isolated corrective acts.

That said, the findings diverge from some earlier conclusions regarding learner affect. For example, Manhood (2017) found that delayed written feedback sometimes generated negative emotional responses in EFL learners, especially when it lacked clarity or personalization. However, the structured and rubric‐supported feedback in the current study may have mitigated such effects by providing clear performance criteria, consistent with feedback design recommendations by Winston and Carless (2020).

Despite these positive outcomes, some limitations must be acknowledged. This study did not fully control for potential confounding variables such as class dynamics or instructor variability. The same instructor delivered feedback across all groups, and while efforts were made to standardize delivery, subtle differences in tone or rapport may have influenced engagement levels (Guilloteaux 2016; Vattøy and Smith 2019). Future research could address this by employing multiple instructors and using blinded delivery protocols.

In addition, while this study focused on written feedback in a controlled classroom context, the effects of feedback timing might differ in digital or asynchronous environments where learners interact with feedback in varied temporal and spatial contexts (Harasim 2012; Robinson 2024; Zygouris‐Coe 2012). Further exploration into modality effects and learner agency across learning platforms is warranted.

## 6 Conclusion and Implications

In conclusion, this study investigated the nuanced effects of immediate and delayed feedback on the engagement and willingness of EFL learners to participate in collaborative learning. The findings revealed that both types of feedback significantly influenced cognitive, affective, and behavioral engagement, highlighting the importance of feedback timing in shaping learner experiences and outcomes. Immediate feedback was particularly effective in supporting cognitive processing and emotional involvement, suggesting that timely correction reinforces learner focus and conceptual clarity. Delayed feedback, while less effective for cognitive and affective dimensions, appeared equally or more beneficial in promoting behavioral engagement and willingness to collaborate, likely due to its role in fostering reflection and autonomy.

The disparities in feedback effectiveness underscore the importance of aligning feedback timing with instructional objectives. Feedback delivered close to the point of instruction enhances procedural learning and reinforces input before errors become entrenched. Conversely, delayed feedback may support higher‐order thinking and long‐term retention, particularly when learners are encouraged to re‐evaluate their performance independently or with peers.

These findings suggest a differentiated approach to feedback implementation, where timing is intentionally varied based on target learning outcomes. For instance, immediate feedback may be prioritized during skill acquisition and practice‐based tasks, whereas delayed feedback could be more effective in collaborative, reflective, or assessment settings.

Notably, the use of Skyroom as the instructional platform introduces implications for feedback delivery in online versus face‐to‐face contexts. In virtual settings, where delayed interaction is often unavoidable, educators may leverage structured delayed feedback to promote deeper processing and sustained learner autonomy. However, synchronous tools can also support immediate corrective feedback in real‐time online classes, preserving the benefits observed in face‐to‐face settings. Future studies should investigate how technological affordances influence the perceived value and cognitive impact of feedback timing in blended and online formats.

From a pedagogical standpoint, teacher training programs should incorporate instruction on how to design and sequence feedback according to learners' engagement profiles and the affordances of digital platforms. Professional development should equip teachers with strategies to balance immediacy, reflection, and learner autonomy when responding to student output.

In terms of curriculum design, feedback strategies should be integrated intentionally, taking into account timing, task type, and learner readiness. Explicit guidelines for both written and oral feedback—embedded within digital and in‐person instruction—can support consistent learner engagement across modalities.

In sum, this study contributes to the broader discourse on feedback efficacy by demonstrating that timing significantly mediates learner engagement and collaboration outcomes. Future research should continue to explore how feedback timing interacts with learner variables such as proficiency, motivation, and digital literacy to inform evidence‐based practices in diverse instructional contexts.

## Conflicts of Interest

The author declares no conflicts of interest.

## Data Availability

The data that support the findings of this study are available on request from the corresponding author. The data are not publicly available due to privacy or ethical restrictions.
